# Evolutionary Dynamics between Phages and Bacteria as a Possible Approach for Designing Effective Phage Therapies against Antibiotic-Resistant Bacteria

**DOI:** 10.3390/antibiotics11070915

**Published:** 2022-07-07

**Authors:** Mahadi Hasan, Juhee Ahn

**Affiliations:** 1 Department of Biomedical Science, Kangwon National University, Chuncheon 24341, Gangwon, Korea; 202216366@kangwon.ac.kr; 2 Institute of Bioscience and Biotechnology, Kangwon National University, Chuncheon 24341, Gangwon, Korea

**Keywords:** phage resistance, trade-off, superinfection exclusion, restriction modification, abortive infection, CRISPR–Cas

## Abstract

With the increasing global threat of antibiotic resistance, there is an urgent need to develop new effective therapies to tackle antibiotic-resistant bacterial infections. Bacteriophage therapy is considered as a possible alternative over antibiotics to treat antibiotic-resistant bacteria. However, bacteria can evolve resistance towards bacteriophages through antiphage defense mechanisms, which is a major limitation of phage therapy. The antiphage mechanisms target the phage life cycle, including adsorption, the injection of DNA, synthesis, the assembly of phage particles, and the release of progeny virions. The non-specific bacterial defense mechanisms include adsorption inhibition, superinfection exclusion, restriction-modification, and abortive infection systems. The antiphage defense mechanism includes a clustered regularly interspaced short palindromic repeats (CRISPR)–CRISPR-associated (Cas) system. At the same time, phages can execute a counterstrategy against antiphage defense mechanisms. However, the antibiotic susceptibility and antibiotic resistance in bacteriophage-resistant bacteria still remain unclear in terms of evolutionary trade-offs and trade-ups between phages and bacteria. Since phage resistance has been a major barrier in phage therapy, the trade-offs can be a possible approach to design effective bacteriophage-mediated intervention strategies. Specifically, the trade-offs between phage resistance and antibiotic resistance can be used as therapeutic models for promoting antibiotic susceptibility and reducing virulence traits, known as bacteriophage steering or evolutionary medicine. Therefore, this review highlights the synergistic application of bacteriophages and antibiotics in association with the pleiotropic trade-offs of bacteriophage resistance.

## 1. Introduction

Since the discovery of penicillin by Alexander Fleming in 1928, antibiotics have saved millions of lives [1]. However, at the same time, bacteria have evolved antibiotic resistance, exposing the limitation of this magic medicine to treat bacterial infections [2]. The emergence and spread of antibiotic-resistant bacteria have been accelerated due to antibiotic misuse and overuse in medicine and agriculture [3,4]. A large number of antibiotic-resistant bacteria have been identified as high-priority pathogens, including methicillin-resistant *Staphylococcus aureus* (MRSA), extended-spectrum β-lactamases (ESBL) producing *Enterobacteriaceae*, vancomycin-resistant enterococci (VRE), and multidrug-resistant *Pseudomonas aeruginosa*, *Streptococcus pneumonia*, *Mycobacterium tuberculosis*, and *Acinetobacter baumannii* [5,6,7,8,9,10]. Human health care, animal industries, food manufacturing companies, and agricultural sectors are facing the antibiotic resistance threat [11,12]. Along with economic losses, antimicrobial resistance tolls 700,000 lives every year throughout the world due to therapeutic failure [13,14]. Bacteria can rapidly acquire molecular mechanisms to evolve antibiotic resistance through horizontal gene transfer (HGT) from everywhere [15]. The infections caused by multidrug-resistant (MDR) pathogens are difficult to treat because of the limited chemotherapeutic options, which has become a top public health concern worldwide. The frequent antibiotic treatment failures have urged the development of alternative therapeutic agents against MDR pathogens [16]. 

Bacteriophages (phages) are the most abundant biological entities that specifically infect bacteria [17]. Phages contribute to the diversity of bacterial communities in terms of the coevolutionary fitness dynamics [18,19]. Recently, phages have gained revived attention as alternative antibacterial agents over conventional antibiotics [20,21,22,23]. In Europe, phages have been used for therapeutic and prophylactic purposes, with a less adverse effect on normal microbial flora and no side effects [22,24]. However, the emergence of phage resistance still remains a major drawback in therapeutic applications [25,26,27]. The emergence of phage resistance is a major concern for the use of phage therapy in terms of the coevolutionary arms races between phages and bacteria [28,29]. The competitive interactions between phages and bacteria are evolutionary processes, ongoing defense strategies and counterstrategies, for survival in nature [20]. Bacteria evolve phage resistance under selection pressure, resulting in the coincidental changes in bacterial fitness and virulence [4,30]. In this context, it may be assumed that phage-resistant bacteria induce cross-resistance to different phages and other antibiotics in the same way as antibiotic resistance. However, there is still little information with regard to the evolutionary trade-offs and trade-ups between phage resistance and antibiotic resistance. Here, we review the antiphage defense mechanisms in association with the changes in fitness and virulence, specifically antibiotic resistance, in bacteria. 

## 2. Phage Structure and Life Cycle 

Phages are estimated to outnumber bacteria by tenfold [31]. Phages were discovered in 1917 and first used as therapeutic agents in 1919 [32,33]. However, the interest in phage therapy was shifted to antibiotics commonly used to treat bacterial infections from the 1940s onwards. Many phages consist of a head/protein capsid containing the genomic material and tail fibers acting as receptor-binding sites [34]. Most phages have polyhedral capsids, predominantly icosahedral, except for filamentous ones [35]. The structure of tailed phages consists of an icosahedral head and a tail with receptor-binding proteins (RBPs) such as tail spikes and tail fibers at the distal end [36,37]. Phage genomes containing single-stranded or double-stranded RNA or DNA are encased in the protein capsid. RBPs are observed in *Myoviridae* as long and short fibers attached to the contractile tail, in *Podoviridae* as spikes or fibers attached to a short non-contractile tail, and in *Siphoviridae* as baseplates, fibers, spikes, or single straight filaments attached to a long non-contractile tail [38]. The key component of the tail is the contractile sheath. In T4 phages, the contractile sheath contracts to less than half of its original length during the infection to insert the tail tube through the outer membrane for genome delivery [39]. 

Phages are completely dependent on their hosts for multiplication. Phages have two distinctive life cycles, lytic and lysogenic. In the lytic life cycle, the phages infect the host, multiply inside, and lyse the host cell to release the mature progeny phages. In the lysogenic life cycle, on the contrary, phages integrate their genome into the host’s chromosome, and integrated phage DNA is replicated concurrently with bacterial DNA. In addition, certain temperate phages are maintained as episomal components rather than being incorporated into the host chromosome. The phage DNA integrated into the host cell is termed as the prophage. However, stresses such as UV radiation, antibiotics, pH, temperature, and water activity can activate prophages [32,40]. When prophage activation is triggered, an irreversible transformation from lysogeny to the lytic life cycle takes place and the phage completes the lytic cycle by making copies and lyses the cell to burst them out [41]. The phage attachment to the bacterial host commences with the binding of RBP on the tip of the phage tail to a target receptor on the host cell surface, occurring in three steps—initial contact, reversible adherence and irreversible attachment [42,43]. The initial step is the random collision of the phage with the hosts and the recognition of the receptors on the host surface [42]. After phage receptor recognition, the phage RBP reversibly binds with the receptor [44]. The last step is the irreversible phage binding to the receptor [45]. After the permanent binding with the receptor, the phage ejects the genetic material into the host cytoplasm [46] and the phage genome is expressed to produce virion particles such as head, tail, base plate, and fiber, followed by simultaneous assembly. After the phage assembly, phage-encoding enzymes such as endolysin and holin help release the progenies from the host cells [47].

## 3. Coevolutionary Dynamics of Phage-Bacteria Interactions 

**Figure 1:**
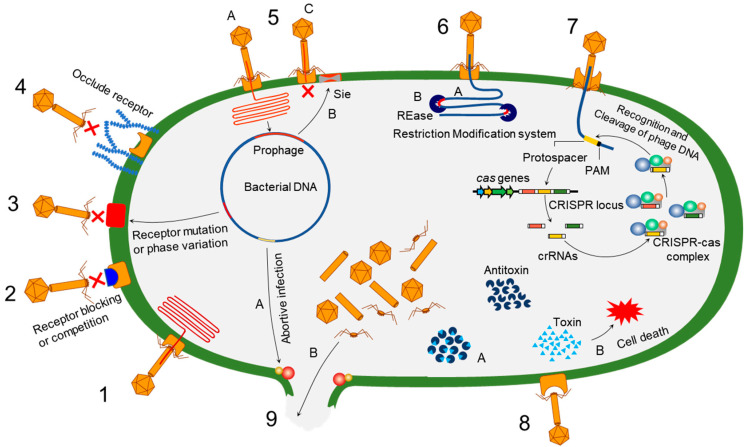
Overview of bacterial antiphage defense mechanisms. 1. Phage infection: successful phage attachment and infection; 2. competition in receptor binding: blocking of the phage-binding receptor to evade phage attachment; 3. alteration in cell surface receptor: mutation or phase variation in the receptor to avoid phage attachment; 4. hiding phage receptor: production of extracellular polysaccharides to hide phage receptor; 5. superinfection exclusion—A: integration of prophage into host genome; B: expression of protein to block DNA entry; C: exclusion of superinfection; 6. restriction-modification system—A: recognition of restriction site; B: cleaving of inserted phage DNA; 7. CRISPR–Cas immunity; 8. toxin–antitoxin immunity—A: antitoxin neutralizes toxin before phage infection; B: phage infection-mediated liberation of toxin to induce reduced metabolism or cell death; 9. abortive infection—A: phage infection mediates expression of abortive infection mechanism; B: release of unassembled phage particles from the host cell.

Bacteria evolve phage resistance through bacteria defense systems under phage selection pressure [48,49] (Figure 1). In this context, phages, however, evolve counter adaptations against bacterial antiphage mechanisms [27,50]. Therefore, phages and bacteria can undergo continuous coevolutionary processes involving phage infection and antiphage defense mechanisms [21,27,50,51,52]. The phage resistance mechanisms of bacteria include non-specific adaptations and specific adaptation systems [48,49]. The non-specific bacterial defense mechanisms (innate immune systems) against phages include the inhibition of phage attachment to the host surface receptors, the prevention of phage genome entry into the host cells, the restriction of secondary phage infection (superinfection exclusion), the activation of endonucleases and methyltransferases (restriction-modification system), and the induction of suicide in infected cell (abortive infection system) [48]. The phage-specific bacterial defense mechanisms (adaptive immune systems) are a second line of antiphage defense systems such as clustered regularly interspaced short palindromic repeats (CRISPR)–CRISPR-associated (Cas) proteins [53]. The phage-resistant bacteria result in phenotypic and genotypic changes, including growth rate, membrane permeability, capsular polysaccharide (CPS) production, phage-binding receptor, virulence, and antibiotic susceptibility [54,55]. The antiphage mechanisms in bacteria developed against phage infection stages, including adsorption, penetration, synthesis, assembly, and release [56,57,58,59].

## 4. Antiphage Defense Mechanisms 

### 4.1. Phage Binding-Based Defense Mechanisms

The first stage of the phage infection process is the attachment to bacterial surface receptors such as lipopolysaccharides (LPSs), pili, outer membrane proteins (OMPs), and efflux pumps [60]. The attachment-blocking mechanism is the predominant defensive strategy for preventing phage infection [59,61].

#### 4.1.1. Prevention of Phage Attachment and Entry

Phage adsorption is blocked by modifying cell surface receptors, producing extracellular polysaccharides, or synthesizing the analogs of receptor-binding proteins, resulting in phage resistance [22,49,62]. To be specific, the mutation in the cell surface receptors, such as pili, flagella, outer membrane proteins, and LPS, is pivotal for the inhibition of phage binding to the host bacteria [63,64,65,66]. The modification of phage-binding receptors occurs in nutrient-rich conditions, while the CRISPR–Cas system is active in nutrient-depleted conditions [66]. The overproduction of glycocalyx, including capsule, slime layer, and exopolysaccharide (EPS), prevents phage adsorption by masking the phage-binding receptors [67,68]. Biofilm cells enclosed in physical barriers, mainly the EPS matrix, confer a high level resistance to phages [69]. An outer membrane lipoprotein (TraT) encoded by the F plasmid overlays phage receptor (OmpA) and prevents the attachment of OmpA-specific phages [70,71]. Mutation in phage-binding receptors can induce bacterial resistance to phage infection. The outer membrane protein OmpA of Escherichia coli K-12 is used as a receptor by T-even-like bacteriophages [72]. Phage resistance is achieved by point mutations, rearrangements, and insertions that modify the OmpA surface protein [73]. 

Bacteria often use a phase variation mechanism, in which bacteria undergo reversible phenotypic changes through alterations in definite loci of the genome, to evade phage infection [74]. Hemophilus influenzae adopts a phase variation mechanism to alter lipooligosaccharide (LOS) construction by changing the lic2A gene, which affects LOS biosynthesis, to resist HP1c1 phage adsorption [75]. Similarly, Salmonella Typhymurium harbors a gtrABC1 cluster which glycosylates the O12 antigen to evade the SPC35 phage infection. Although BtuB is the receptor of phage SPC35, it is O12 antigen which facilitates the adsorption with BtuB. Upon the selection of phage SPC35, gtrABC1 glycosylates the O12 antigen, which successfully prevents the phage infection [76]. Random variation in hypermutable homonucleotide 7–11G (polyG) tracts causes phase variation in Campylobacter and in Campylobacter jejuni. The polyG tracts were observed in 20–30 genes, which are mostly related to the modification of surface structures [77,78]. The Campylobacter jejuni strain was reported to alter its phase by changing the number of Gs in the hypervariable gene cj1421, which codes for GlafNAc MeOPN transferase, to become resistant to phage F336. The resistant isolate lacked the O-methyl phosphoramidate moiety as it was apparent from the periodate treatment that the carbohydrate moiety was the receptor of phage F336 [78]. The alteration of the number of Gs was also observed in the C. jejuni genes cj1139 and cj0039 [77]. Phase variation also modulates the mutual persistence of phage and bacterial host. When bacteroides intestinalis was selected with crAss-like phage crAss001, the bacterial host developed resistance via a phase variation in CPS [79].

The outer membrane vesicles (OMVs) limit the access of phages to the surface. The OMV successfully protects *P. aeruginosa* against two phages, KT28 and LUZ7 [80]. Phages also face competition from receptor molecules for binding with bacteria. Microcin J25 (MccJ25), a 21 L-amino acid plasmid-encoded antimicrobial peptide, binds with an outer membrane receptor of T5 phage, FhuA [81]. The antimicrobial peptides, as analogs, competitively bind to phage-binding receptors such as FhuA [62]. Thus, MccJ25 prevents the T5 phage binding with FhuA and infect the host [62].

#### 4.1.2. Superinfection Exclusion Systems

The prophage integrated in a host chromosome blocks the entry of the phage genome, known as superinfection immunity [82]. The entry of the phage genome into the host cell is regulated by superinfection exclusion (Sie) [83]. The pre-existing Sie-related genome encoded in the prophage plays an important role in phage–phage interactions [59]. The Sie system encoded by Imm and Sp prevents phage genome injection into the host cells. Imm blocks the passage of the phage genome through the plasma membrane, and Sp protects the bacterial murein layer which is a target site for tail-associated lysozymes (gp5) [84,85]. Spackle, a periplasmic protein, triggers the Sie system and inactivates gp5 through the formation of a stoichiometric complex [86]. Twitching inhibitory protein (Tip), expressed by the *P. aeruginosa* prophage D3112, prevents type IV pili expansion by interacting with ATPase PilB, leading to the inhibition of phage infection [87]. Lambdoid phages, including mEp213, mEp237, and mEp410, cannot infect mEp167 lysogenic cells [88,89]. Subcluster B2 Hedgerow and Rosebush phages bind to the receptors in a Wag31 kinase-dependent manner [90]. A Cluster F mycobacteriophage Fuitloop-encoded protein gp52 interacts with Wag31, resulting in the prevention of superinfection [91]. Similarly, Cor protein blocks the phage-binding receptor (FhuA) and mediates superinfection exclusion [89]. The Cor protein also interacts with the outer membrane proteins such as OmpA, OmpC, OmpF, OmpW, LamB, and Slp, which can block the attachment of FhuA-dependent phages [92]. The expression of the cI repressor in lysogenic cells promotes the cleavage of the infecting phage genome, leading to the development of the antiphage defense system [25,93].

### 4.2. Inhibition of Phage Synthesis and Assembly

After phage adsorption, the phage genome enters the host cell. The injected phage genome is degraded at the levels of replication, transcription, translation, and assembly by restriction-modification (R-M) and CRISPR–Cas systems [94,95].

#### 4.2.1. Restriction-Modification Systems

Restriction-modification (R-M) systems are prokaryotic immune systems used to protect bacteria against foreign DNA such as phages and plasmids. The R-M systems consist of restriction endonucleases (REases) and methyltransferases (MTases), which degrade unmethylated phage genomes and methylate host DNA to protect the self-genome from cleaving by REase, respectively [96,97,98]. The REases are classified into four types according to their subunit structure, recognition site, cofactor requirement, and specific activity [99]. Type I, II, and III endonucleases correspond to the MTases with the same reignition sites. Type I R-M systems are composed of three different subunits required for restriction, methylation, and recognition. Type II and Type III R-M systems are composed of two distinct subunits for restriction and modification. Unlike other R-M systems, Type IV R-M systems are composed of a single unit that can cleave modified sequences such as methylated hydroxymethylcytosine (HMC) and other modified bases [100]. Homing endonucleases are involved in specific DNA modifications [101]. The hemi-methylated molecules are targeted for methylation by MTase, and completely methylated DNA is excluded from both of these functions [102]. REase cleaves the unmethylated DNA into benign segments after the successful attachment of a phage and insertion of DNA into the host cell. Bacteriophage exclusion (BREX) does not degrade unmethylated DNA but inhibits the replication of the phage genome in the host cells [103].

#### 4.2.2. CRISPR–Cas Systems

The CRISPR–Cas systems are responsible for adaptive immunity, which have the ability to effectively destroy the injected phage genome by remembering past infections [59,95]. The CRISPR–Cas locus is composed of short identical repeats interspaced with space sequences which are not identical and flanked by Cas protein genes. The repeats are identical in length and sequence, whereas the spacers are uniform only in length. Repeat lengths vary from 21 bp to 47 bp based on the species. Spacers, or protospacers, are also similar in size, between 20 and 72 bp [104]. Protospacer adjacent motif (PAM) (2–5 bp) is used to identify the target DNA. This short-conserved region is essential to differentiate between self- and non-self-DNA [105]. After the recognition of PAM, protospacer, a segment of downstream DNA, is copied from the foreign DNA and transcribed into CRISPR RNAs (crRNAs). The degradation of foreign DNA or RNA occurs when crRNA with trans-activating crRNA guides the Cas9 endonuclease to the cleaving site of the invading DNA [106,107]. Bacteria evolve CRISPR–Cas-mediated phage resistance through several steps, including the active integration of phage-derived proto-spacers into the CRISPR loci, the transcription of CRISPR loci to express precursor-CRISPR RNA (pre-crRNA), the modification of pre-crRNA into short crRNA, the formation of guide RNA (crRNA and tracrRNA), and the RNA-guided interference of the target phages (sequence-specific immunity) [108,109,110]. The CRISPR–Cas systems are one of the major antiphage defense strategies, showing more than 70% Cas positivity in phage-resistant bacteria [111,112,113].

#### 4.2.3. Abortive Infection Systems

Abortive infection (Abi) systems can block the replication, transcription, and translation in phage-infected cells, which protect adjacent uninfected cells within the population from phage attack [49,114]. Phage infection is very specific; thus, Abi protects only very closely related bacteria by killing only the infected host [115]. The functional modules are required for the Abi system to recognize the phage infection and shut down the metabolism [114]. The cell killing module is initiated by the recognition of phage infection. However, this module is regulated very tightly so that the Abi system does not activate unless the cell is infected and hinders normal cell activity [61]. 

One of the most studied Abi is the Rex system, which is found in the λ phage of lysogenic *E. coli* strains [59]. Rex is a two-component system that aborts the lytic growth of phages expressed by λ prophage genes, *rexA* and *rexB* [116]. A protein–DNA complex is produced as a result of replication or recombination during the phage infection that activates RexA [117]. One copy of RexB, a membrane-anchored protein with four transmembrane helices, is activated by two copies of RexA, showing that the protein ratio is critical for Abi action [118]. RexB produces an ion channel in the inner membrane, leading to a severe loss of membrane potential and a subsequent drop in cellular ATP levels and resulting in a decrease in the synthesis of macromolecules, consequently inhibiting bacterial growth and aborting phage infection [116]. Although the activation of Rex kills the majority of cells, it protects about 1/100 of the “Rex-activated” population, leading to the theory that the Rex system causes an osmotic shift that induces a stationary phase that deters the propagation of a superinfecting phage such as T4 [119,120].

DNA replication, transcription, translation, the packing and assembly of phage particles, and host lysis are inhibited by Abi systems [121]. Abi systems induce altruistic self-destruction in phage-infected host cells through toxin–antitoxin (TA) systems [122,123]. The replication of phage DNA is inhibited by the phage exclusion system and disruption of replication-related genes [103,124]. The transcription and translation of the phage genome are also inhibited by the disruption of regulatory proteins [124,125]. Abi systems block phage assembly and induces the death of phage-infected host cells through the TA system, leading to the inhibition of phage replication [123,126,127]. Phage packaging interference (Ppi) proteins play an important role in interfering with the phage packaging [128].

#### 4.2.4. Toxin–Antitoxin Systems

TA systems consist of two genes, a toxin gene and toxin-diminishing antitoxin gene [129]. Toxins inhibit major cellular processes, including replication, translation, and cell wall construction, while antitoxins neutralize the cognate toxins [61]. Toxins impact DNase and RNase activities, ATP synthesis, and replication inhibition, as well as cell division inhibition [61]. The TA systems are involved in essential biological functions such as growth arrest, survival, biofilm formation, plasmid integrity, phage resistance, and virulence [96,130]. Eight different types of TA systems (types I-VIII) are classified based on the characteristics of the toxin and antitoxin molecules (protein or RNA) and the toxin neutralization reactions [131]. TA-encoding genes are found in bacterial chromosomes, plasmids, and phage genomes, particularly in prophages [132].

RnlAB is a type II TA system identified in *E. coli* K12 that provides effective protection against phage T4 with a defective *dmd* gene [133,134]. The *rnlA*-encoded endoribonuclease RNase LS was first known as a bacteriophage T4 infection inhibitor, cleaving T4 mRNAs to limit the expression and preventing T4 phage multiplication [133,134]. The toxin-encoding *rnlA* is responsible for RNase activity and the *rnlB* encoded downstream of *rnlA* encodes antitoxin RnlB which neutralizes the toxicity of RnlA [133,134]. The RnlB is readily degraded after infection with a T4 *dmd* mutant [133]. The host transcription is halted by T4 infection due to *rnlAB* transcription, leading to the release of active RnlA endoribonuclease [132,133].

## 5. Phage-Evolving Counterstrategies 

Despite the development of bacterial defense mechanisms, phages can evolve counterstrategies to evade bacterial antiphage defense systems through fast adaptive plasticity and replication, including point mutations, genomic rearrangements, and inactivating proteins critical for antiphage defense mechanisms [135].

### 5.1. Counterstrategies against Receptor Alterations

Phages can evolve new infection mechanisms to evade adsorption-blocking-dependent phage-resistance systems by recognizing new or altered receptors on the host cell surface. Phages can modify their tail fibers (receptor-binding sites) to cope with antiphage system acquired by the conformational changes in the phage-binding receptors [136,137]. The tail fiber protein J of λ phages binds to the outer membrane protein (LamB) of *E. coli* for infection. The λ phages evolve the J gene that can bind to an alternative receptor (OmpF) instead of modified LamB [136]. Although pathogenic *E. coli* modifies lipopolysaccharide biosynthesis and/or membrane transporter protein (OmpA) to resist phage infection (ϕHP3), the putative tail spike protein of ϕHP3 is altered to avoid the antiphage defense system [138]. In addition, phages produce enzymes such as depolymerases and hydrolases that degrade exopolysaccharides, leading to an increase in phage adsorption rates [49,139]. The phage-encoding enzymes are classified into lyases and hydrolases based on the cleavage of polysaccharides [59]. A tail spike protein of the *Proteus* bacteriophage (PmiS_PM-CJR) encodes pectate lyase to degrade the biofilm matrix [140]. Similarly, a tail protein of the phage IME200 degrades the capsule polysaccharide, in *Acenetobacter baumanni* [141]. *Acinetobacter* podo phage Petty encodes depolymerase which degrades capsular EPS [142]. *Pseudomonas aeruginosa* and *Bordetella* phages create phenotypically diverse phages through hypermutable polyG tracts in order to overcome phage resistance [143,144].

### 5.2. Counterstrategies against Restriction Modification

Phages also develop counterstrategies against the restriction-modification systems [49,145]. The phage counterstrategies include the alteration of restriction sites within the phage genome, the degradation of cofactors required in the restriction-modification systems, and the methylation of the phage genome [49,59,98]. To evade the R-M systems, phages change the restriction recognition site through point mutation or DNA modification. The T4 phage’s genome contains HMC rather than cytosine so that the REase enzyme may not detect the restriction site of the T4 phage [146]. Similar nucleotide modifications occur in *Salmonella* phage Vil (thymidine replacement by 5-(2-aminoethoxy)methyluridine), *Pseudomonas* phage M6 (thymidine replacement by 5-(2-aminoethyl)uridine), *Bacillus* phage SP8 and SPO1 (5-hydroxymethyl uridine (5hmdU)), and cyaophage S-2L (diaminopurine substituted for adenine) [147,148,149]. Some phages containing MTase can protect the phage genome from bacterial endonuclease cleavage [150]. The phage KP15 contains DNA adenine methyltransferase (Dam) and DNA cytosine methyltransferase (Dcm) [150,151]. Coli phages (T2 and T4) and lactococcal 936-type (Phi93, Phi145, and Phi15) encode the methyltransferase gene [150]. The glycosylated HMC of the phages T4, T2 and T6 is degraded by glucose-modified restriction (GMR) proteins such as GmrS and GmrD in *E. coli* CT596 [152,153]. However, the GRM enzymes are inactivated by T4 phage internal protein I (IPI*), which is degraded by the GMR fusion protein in uropathogenic *E. coli* UT189 [154]. The phage P1 has Dar proteins (DarA and DarB) which defend the phage genome from the type I RM system. Structural analogs of specific phage DNA sequences protect the phage genome against R-M systems by sequestering it [155].

### 5.3. Counterstrategies against CRISPR–Cas

Phages can evade CRISPR–Cas systems through point mutation in the protospacer region and the expression of anti-CRISPR proteins [110,156]. To circumvent CRISPR–Cas-mediated immunity, the point mutations in the CRISPR–Cas-targeted sequence and the formation of nucleus-like structures prevent recognition and the cleavage of target sites [157]. The anti-CRISPR proteins directly inactivate the CRISPR–Cas effector complex. Phages need more than one anti-CRISPR defense mechanism to circumvent a CRISPR–Cas system because of the presence of several different CRISPR spacers [65]. Phages successfully infect *P. aeruginosa* containing type I-F CRISPR–Cas systems. The anti-CRISPR proteins, including AcrF1, AcrF2, AcrF3, AcrF4, and AcrF5, inhibit CRISPR–Cas-mediated gene editing [158]. The *Listeria* phage (ϕLS46) encodes anti-CRISPR protein (AcrVIA1) to inhibit the type VI-A CRISPR–Cas system of *Listeria seeligeri* [159]. The T4 phage can repair its genome cleaved by a CRISP–Cas system by using phage-encoded recombinase UvsX [160]. The temperate phage ΦAP1.1 expresses AcrIIA23, an anti-CRISPR protein, to cease the functionality of Cas9. The inhibition of Cas9 activity allows ΦAP1.1 to integrate into direct repeats of CRISPR and neutralizes the immunity. The loss or alteration of spacers modulates the type II-A CRISPR immune response during integration or excision cycles [161].

### 5.4. Counterstrategies against Abortive Infection

Interestingly, phages induce mutations in their own genome and produce antitoxin proteins to evade abortive infection systems [127]. The T4 phage interferes with the Rex antiphage mechanism to successfully complete the phage replication cycle and produce phage virions in the presence of ATP-dependent and host inner membrane-associated proteins (RIIA and RIIB) [162,163]. T4 phages form plaques on *E. coli* K-12 strain with lysogenic λ in the presence of the rII locus encoding rIIA and rIIB genes, whereas T4rII mutants are unable to form plaques [163]. Phages can also evade the Abi caused by the PifA protein through mutations in the phage proteins gp10 and gp1.2 [164]. PifA is a membrane-associated protein encoded by the F plasmid *pifA* gene which suppresses the transcription of T7 late genes [165]. PifA interacts with the membrane integrity to promote the loss of ATP without lysing and killing the cells [166]. The T7 proteins gp10 and gp1.2 activate the PifA abortive mechanism [167].

## 6. Coevolutionary Trade-Offs between Phage Resistance and Antibiotic Resistance 

The evolutionary interactions between phages and bacteria result in the emergence of phage resistance that positively or negatively compromises the antibiotic resistance [168,169]. Phage therapy can enhance not only the resistance to phages but also the susceptibility to antibiotics [30,170]. The bacteria that evolve phage resistance may make trade-offs with bacterial growth, virulence, antibiotic resistance, nutrient uptake, and biofilm formation [27,171]. The enhanced antibiotic susceptibility, which is the positive effect of phage resistance, is a possible solution to overcome the limitations of phage therapy [15] (Table 1). 

### 6.1. Phage-Binding Receptor-Mediated Trade-Offs

The emergence of phage-resistant bacteria is associated with alterations in phage-binding receptors [184]. The bacterial cell surface components are involved in various cellular processes, including nutrient transport, bacterial motility, biofilm formation, and biosynthesis, which incur fitness costs [27]. Bacteria developing resistance to phages reduce virulence because the conformational changes in phage-binding receptors impose high fitness costs [185]. *Klebsiella pneumoniae* ST258 evolves resistance to the phages Pharr and KpNIH-2 due to the mutation in *galU* which regulates cell envelope synthesis, galactose metabolism, and trehalose metabolism. Thus, this phage-resistant *K. pneumoniae* ST258 results in a significantly low growth rate [172]. The resistance of *Acinetobacter baumannii* to the phages ΦFG02 and ΦCO01 is due to the deletion of *gtr29* and *gpi*, respectively. Mutations in the genes *gtr29* and *gpi* cause reduced biofilm formation, antibiotic resistance, and fitness because these genes are responsible for the biosynthesis of CPSs [173]. The alterations in virulence factors such as CPS and LPS are caused by mutations in genes (*wzc*, *wbaP*, and *wcaI*) related to the biosynthesis of CPS and LPS, which are responsible for phage resistance and virulence attenuation [25,186,187]. The change in the CPS that mediates protection against β-lactams improves the access to antibiotics, leading to enhanced antibiotic susceptibility [188,189]. The alteration in the LPS that mediates membrane permeability promotes antibiotic susceptibility [4,190]. The fitness loss of the PAO1 mutant *P. aeruginosa* is attributed to the modification of LPS and T4p receptors, which are responsible for the regulation of cell metabolism [174]. The outer membrane protein (FhuA) is the receptor for phages such as T1, T5, Φ80, N15, and HK022, as well as mEp167, mEp213, and mEp450 coliphages [92,191,192,193]. The alteration in FhuA results in phage-insensitive mutants and an enhanced susceptibility to rifamycin, albomycin, colicin M, and microcin J25 [193,194]. Therefore, the modification or loss of bacterial cell surface molecules promotes phage resistance and induces phenotypic conversion [27]. The phage resistance can trade-off or trade-up against virulence, fitness, and antibiotics resistance [64].

### 6.2. Efflux Pump-Mediated Trade-Offs

The bacterial cell surface molecules include virulence factors and efflux transporters, which are involved in adherence, secretion, and uptake [195,196]. Bacteria have membrane-associated efflux pumps to secrete antimicrobials, metabolites, and quorum-sensing signal molecules [197,198]. The major efflux system consists of a periplasmic lipoprotein (AcrA), inner membrane transporter (AcrB), and outer membrane protein (TolC) in Gram-negative bacteria [199,200]. The efflux pumps play a major role in antibiotic resistance and bacterial pathogenicity [201]. The *tolC*-deficient *E. coli* was more sensitive to antibiotics and bile salts than the wild-type strain [202]. The phage-resistant bacteria are deficient in drug efflux pumps, resulting from the evolutionary trade-offs [30,203]. The phage-binding receptors function as multidrug efflux pumps such as TolC and OprM [189]. Modifications in TolC as a receptor for *Escherichia* phage (TLS) and Salmonella phages (ST27, ST29, and ST35) can enhance susceptibility to antibiotic substrates [170,204]. Alterations in porins, which are phage-binding receptors, are associated with the reduction in antibiotic resistance in phage-resistant bacteria [30,205]. The genomic deletions in antibiotic biotic-resistant bacteria are induced by PIAS, leading to compromised efflux pump systems [20,206]. The loss of outer membrane- and efflux-associated antibiotic resistance is attributed to the modification of phage-binding receptors such as MexAB, MexCD, MexEF, and MexXY [20]. The Mex efflux pump system consists of an inner membrane transporter (MexB, MexY), periplasmic membrane proteins (MexA, MexX), and an outer membrane protein (OprM) [207,208]. The outer membrane permeability-associated low uptake and efflux pump-associated active transport contribute to antibiotic resistance in bacteria [209,210]. Quorum sensing downregulates the phage-binding receptors (OmpK) [187,211]. The efflux pumps impaired by phage resistance can be applied for phage steering to sensitize phage-resistant bacteria to antibiotics [16,30,212].

### 6.3. Fitness Trade-Offs of Antibiotic Resistance

Receptor-mediated phage resistance imposes high fitness costs and is compromised with increased phage resistance, resulting in trade-offs between enhanced phage resistance and reduced fitness traits [54,138]. The compensatory mutations under phage selection pressure contribute to attenuated fitness, reduced virulence, and increased antibiotic susceptibility [26,64,213,214,215]. This phenomenon is a double-edged sword in that one trait can be developed for survival but other traits can be lost by way of compensation [212]. The altered fitness traits in phage-resistant bacteria include decreases in growth rate, motility, nutrient transport, virulence, and antibiotic resistance [4,21,27,28]. Phage-binding receptors are involved in bacterial pathogenesis, including attachment, colonization, invasion, and immune evasion [216]. The phage resistance trade-off with virulence is responsible for capsule production and biofilm formation [28,217,218,219]. The lack of modification of phage-binding receptors can cause virulence attenuation in phage-resistant bacteria [64,220]. However, unlike receptor-mediated phage resistance, CRISPR-mediated phage resistance does not induce trade-offs between phage resistance and virulence [219]. The phage-induced deletion of resistance gene clusters including *bla*, *aph*, *sul*-*fol*, and mph restores the susceptibility of phage-resistant bacteria to β-lactam resistance genes, aminoglycoside, sulphonamide/trimethoprim, and macrolide, respectively [25]. However, phage resistance can pleiotropically not only make trade-offs but also trade-ups with fitness, virulence, and antibiotic resistance [4,30,51,189,215,221,222]. The major negative effects of phage resistance in bacteria include the enhanced production of β-lactamases, resulting in antagonistic pleiotropy [189,215,222]. The pleiotropic trade-ups depend on the structural barriers of OMPs and LPSs, leading to enhanced antibiotic resistance and virulence [23,189]. The substrate-specific uptake channels can enhance the cross-resistance to gyrase inhibitor-associated antibiotics due to the mutations in OmpF and Tsx porins [23,223,224]. The trade-ups between phage resistance and antibiotic resistance are regarded as a main limitation of phage therapy [189,215]. In contrast to the trade-offs, the modification in LPS can trade-up with increased antibiotic resistance in combination with decreased outer membrane permeability [223]. Therefore, the therapeutic application of phages needs to consider both antagonistic and synergistic pleiotropies to effectively control MDR bacterial infections [189].

## 7. Concluding Remarks 

The modification (or loss) of phage-binding receptors on the bacterial cell surface results in resistance towards some phages and susceptibility towards others that have alternative receptors. The trade-offs can sensitize phage-resistant bacteria to antibiotics. Thus, combination chemotherapy can be a potential alternative to treat MDR bacteria and also provide a benefit of reusing conventional antibiotics. The phage cocktails can be used to overcome receptor-mediated phage resistance. The evolved phage-induced antibiotic sensitivity (E-PIAS) can be used to overcome the drawback of phage therapy. The combination therapy of E-PIAS and antibiotics can be an alternative method to effectively inhibit MDR pathogens. In view of evolutionary trade-offs, bacteria developing phage resistance by receptor mutations are re-sensitized to antibiotics due to the gene deletions in the mucoid state. The reduced virulence and enhanced antibiotic susceptibility in phage-resistant bacteria ensure the possibility of using phage–antibiotic combination therapy. Phages need specific receptors such as efflux-, uptake-, permeability-, and maintenance-associated bacterial cell surface molecules on the bacterial cell surface. The modification of phage-binding receptors can lead to the trade-offs with decreased antibiotic resistance. Therefore, the trade-offs in phage resistance play an important role in developing effective phage therapy using positive evolutionary outcomes such as decreased virulence, fitness defects, and enhanced antibiotic susceptibility. Specifically, the trade-off with antibiotic resistance can overcome the negative effect of phage therapy. Phage therapy can be tailored factoring in the consequences of trade-offs, which provides synergy with antibiotics to treat MDR infections. However, the application of phages in the treatment of MDR bacteria has to carefully considered in order to avoid negative evolutionary trade-ups. Future studies need to understand the discrepancy between coevolution dynamics in vitro and in vivo on the interaction of phages and antibiotics.

## Figures and Tables

**Table 1:** Trade-offs between phages and bacteria.

Host	Phage	Phage-Binding Receptors	Antiphage Mechanism	Trade-Off	References
Phage-binding receptor-mediated trade-off					
*Klebsiella pneumonia* ST258	Pharr	Capsular polysaccharide	Mutation in *galU*	Decrease in growth rate	[172]
*Klebsiella pneumonia* ST258	ΦKpNIH-2	LPS, OmpC	Mutation in *galU*	Decrease in growth rate	[172]
*Acinetobacter baumannii*	ΦFG02	Capsule	Defective capsule production	Increase in antibiotic susceptibility and, reduction in biofilm formation	[173]
*Acinetobacter baumannii*	ΦCO01	Capsule	Defective capsule production	Increase in antibiotic susceptibility and reduction in biofilm formation	[173]
*Pseudomonas aeruginosa* PAO1	φKZ, KTN4, LUZ19	Type IV pili	Mutation in T4p and global regulatory genes	Decrease in metabolism	[174]
*Pseudomonas aeruginosa* PA14	Phage DMS3vir	Type IV pili	Loss of pili	Significant competitive cost	[66]
*Pseudomonas aeruginosa* PAO1	KT28, KTN6, LUZ27	LPS	Mutations in LPS and global regulatory genes	Reduction in fitness	[174]
*Escherichia coli*	T5	FhuA	Confrontational changes	Reduction in ferrichrome uptake	[175]
*Shigella flexneri*	A1-1	OmpA	Mutations in *ompA*	Increase in vancomycin sensitivity and loss of intracellular movement	[54]
*Shigella flexneri*	A1-1	LPS	Mutation in genes *gmhA* and *gmhC*	Increase in erythromycin sensitivity and loss of intracellular movement	[54]
*Escherichia coli* SB1004	ΦJE	OmpC	Deficient OmpC	Increase in sensitivity to peptide cecropin D	[176]
*Lactobacillus helveticus* ATCC10386	ΦLh56	S-layer	Point mutation	Decrease in S-layer integrity	[177]
*Lactobacillus delbrueckii* subsp. *lactis* Ab1 T-type	Phage YAB	polysaccharide–peptidoglycan complex	Receptor modification	Decrease in proteolytic and acidifying activities	[43,178]
*Enterococcus faecalis* V583	Phi4	Enterococcal polysaccharide antigen (Epa)	Mutation in *epaX*, *epaAC*, and *epaY*	Increase in susceptibility to cell wall-targeting antibiotics	[179]
*Enterococcus faecalis* SF28073	Phi47		Mutation in *epaR* and *epaS*	Increase in susceptibility to cell wall-targeting antibiotics and deficiency in intestinal colonization	
*Acinetobacter baumannii*	Phab24	Capsule	Mutation in *gtr9*	Decrease in virulence and increase in colistin susceptibility	[15]
Efflux pump-mediated trade-off					
*Pseudomonas aeruginosa* PAO1	OMKO1	OprM	Suppression of *oprM*	Increase in antibiotic sensitivity	[30]
*Pseudomonas aeruginosa* MDR	PIAS	MexXY-OprM	Loss or substantial modifications	Increase in antibiotic sensitivity	[20]
*Escherichia coli*	U136B	TolC	Mutation	Increase in sensitivity to tetracycline and colistin	[4]
Superinfection exclusion-mediated trade-off					
*Pseudomonas aeruginosa*	Pf	Type IV pili	PA0721-PilC complex	Suppression of twitching motility	[180]
*Pseudomonas aeruginosa*	D3112	Type IV pili	PilB-gp05 complex	Inhibition of twitching motility	[181]
*Escherichia coli*	mEp167	FhuA	Antiadsorption (Cor-OMP interaction)	Reduction in ferrichorme uptake	[92]
*Streptomyces coelicolor*	φC31	Glycoproteins	Mutation in *ppm1*	Increase in antibiotic susceptibility	[182]
CRISPR–Cas-mediated trade-off					
*Streptococcus thermophilus*	Phage 2972		Spacer addition	Reduction in fitness	[183]
*Pseudomonas aeruginosa* PA14	Phage DMS3vir	Type IV pili	Spacer addition and loss of pili	Reduction in fitness	[66]
R-M system-mediated trade-off					
*Lactobacillus delbrueckii* subsp. *lactis* Ab1 C-type	Phage YAB	Polysaccharide–peptidoglycan complex	Type I R-M system	Decrease in proteolytic and acidifying activity	[43,178]

## Data Availability

Not applicable.
